# Sex Bias in Systemic Sclerosis: from Clinical to Immunological Differences

**DOI:** 10.1007/s12016-025-09062-1

**Published:** 2025-05-27

**Authors:** Lazaros I. Sakkas, Dimitrios P. Bogdanos, Ian C. Chikanza

**Affiliations:** 1 Faculty of Medicine, School of Health Sciences, University of Thessaly, Larissa, 41 500 Greece; 2 Division of Rheumatology, IASO General Clinic, Larissa, Greece; 3 Department of Rheumatology and Clinical Immunology, Faculty of Medicine, School of Health Sciences, University of Thessaly, Larissa, Greece; 4 Paediatrics Department, University of Zimbabwe, Harare, Zimbabwe; 5 Department of Medicine, Catholic University, Harare, Zimbabwe; 6 International Arthritis and Hypermobility Centre, Harley Street Clinic, London, UK

**Keywords:** Gender, Clinical manifestations, Female, Sex dimorphism, Systemic sclerosis, X-chromosome

## Abstract

Systemic sclerosis (SSc) is a chronic autoimmune disease characterized by microvasculopathy, extensive fibrosis, and autoantibodies. The disease affects mostly the female sex. In this review, we highlight sex bias in clinical manifestations in SSc, and the pathophysiological changes underlying this bias. Male sex is associated with the diffuse cutaneous form of the disease, digital ulcers, interstitial lung disease, and worse prognosis. These clinical differences can be attributed to sex hormones and sex chromosomes, as females differ from males in sex hormones (estrogens in females, androgens in males) and sex chromosomes (XX in females, XY in males). Estrogens in females generally have immunostimulatory and profibrotic effects, and androgens have immunosuppressive effects. The X-chromosome contains many immunity-related genes, but the double dose of X-linked genes in females is avoided by random inactivation of one X-chromosome (XCI). However, many X-linked immunity-related genes, including toll-like receptor (TLR)7, TLR8 and Bruton’s tyrosine kinase (BTK), escape XCI resulting in a biallelic expression with pathophysiological implications. Also, autosomal genes are differentially expressed between sexes. Therefore, sex should be included in future studies on SSc to aid in forming predictive algorithms and helping therapeutic decisions in this difficult-to-treat disease.

## Introduction

Systemic sclerosis (SSc) is a complex systemic disease characterized by microvasculopathy and extensive fibrosis that compromise organ function and increase morbidity and mortality [1]. Patients have many autoantibodies(autoAbs), some of which are disease-specific, such as anti-DNA topoisomerase I (ATA), anticentromere antibodies (ACA), and anti-RNA polymerase III antibodies (ARPA). The disease is usually divided into diffuse cutaneous (dcSSc) and limited cutaneous (lcSSc) subtypes to help predict organ involvement, but autoAbs serve this purpose better [2]. The pathogenesis of the disease is incompletely understood. However, adaptive immune responses with B cells producing profibrotic cytokines and autoantibodies, and T cells with TH2 cells producing profibrotic interleukin(IL)−4 and IL-13 and cytotoxic T cells causing microvasculopathy, are implicated [1, 3]. Prominent adaptive and innate immune signatures were present in early SSc, with adaptive immune signatures being associated with shorter disease duration [4]. Environmental factors appear to be very important in disease development. Immigrants in Sweden who were offspring of Serbian and Russian parents had an increased risk for SSc compared to their parents (OR 3.89), similar to that of inhabitants of Sweden, which points to strong environmental effect [5]. Environmental factors, such as silica, heavy metals, and solvents, have been associated with the development of SSc [6].

SSc has a strong female susceptibility bias, as the female-to-male ratio is up to 17:1 [7]. It has been known for a long time that females mount a stronger immune response to vaccinations relative to males and have increased susceptibility to autoimmune diseases. On the other hand, men are generally more susceptible to infections exhibiting higher incidence and severity [8], and during the recent pandemic of SARS-CoV19, men were more likely to exhibit severe disease and increased mortality compared to women [9–13].

Although ignored for years, it is increasingly recognized now that females differ from males, apart from susceptibility to, in disease manifestations and prognosis in various inflammatory and autoimmune rheumatic diseases [14]. In this narrative review, we searched PubMed using the terms “systemic sclerosis” and “gender” to find gender differences in clinical manifestations and immune responses. We also retrieved references from relevant publications. We aimed to highlight gender bias in clinical manifestations and prognosis in SSc and the pathophysiological changes that underlie this bias. In this review, sex and gender are used interchangeably, although gender has connotations related to personal attitude and social context.

## Clinical Aspects

Recent reports have made it clear that there is a sex bias in clinical manifestations and prognosis for SSc. Apart from disease susceptibility, women develop SSc at younger age than men. The most susceptible age for SSc was 50–69 years in men and 40–59 years in women [15], and male sex was a risk factor for occupational exposure (OR 10.3–19.3) [16–19].

### Skin Tightness

In the international European scleroderma trials and research (EUSTAR) database with 9182 patients, and in patients with early disease, male sex was independently associated with dcSSc (OR 1.68) [20–22]. Furthermore, men with lcSSc had internal organ involvement like men with dcSSc [22]. Similarly, male sex was associated with dcSSc in many large SSc cohorts from diverse ethnic populations [23–30].

### Digital Ulcers

Digital ulcers (DUs) are a frequent manifestation of microvasculopathy in patients with SSc, causing pain and functional limitation, and leading to infections, acro-osteolysis, and impairment of health-related quality of life (HRQoL). In the EUSTAR database, male sex was independently associated with DUs (OR1.28) [20]. Also, male sex was associated with DUs in other cohorts, such as in Chinese patients [24, 31] and other ethnic populations [27, 32, 33] and in an international juvenile SSc cohort [34] and was a risk factor for DUs [35, 36]. On the other hand, female patients had Raynaud’s phenomenon more frequently in the Chinese Rheumatism data center [24].

However, no sex difference in DUs was found in the Canadian SSc group registry [37]. Moreover, in a systematic review, sex did not appear to influence the degree of microvasculopathy, as detected by nailfold capillaroscopy (NFC) in SSc [38].

### Calcinosis

Female gender was associated with calcinosis and self-reported symptoms in French patients with SSc [30], while calcinosis was more common in men than women in African blacks with SSc [39]. Spinal calcinosis was associated with male sex and severe peripheral vasculopathy (DUs and/or acro-osteolysis) in Japanese patients [40].

### Musculoskeletal Manifestations

Arthralgias/arthritis were more frequent in women than men in SSc [24, 39, 41], but the findings on myositis are less clear. Myositis was more frequent in men than women in the EUSTAR database of patients with early SSc [22], and SSc cohorts from different countries [25, 29, 41], but in other SSc cohorts, no sex bias was found [42, 43]. Pain was common in patients, and pain intensity was higher in women compared to men in a multinational study [44]. Frailty, prevalent in SSc patients, showed no sex difference [45].

Women had higher disability score (HAQ-DI) compared to men in three randomized controlled trials (RCTs) of dcSSc [46], and female sex was a risk factor for reduced physical function in a multivariable analysis of an international scleroderma patient-centered intervention network cohort [47].

### Gastrointestinal Tract Involvement

In SSc, the gastrointestinal tract (GIT) is affected in most patients, but the sex difference is less clear. Many patients have difficulties with oral hygiene due to small oral aperture, but the risk for periodontitis was higher in male than female patients [48].

Some studies found that men, compared to women, had an increased frequency of GIT involvement [28] and more likely to receive blood transfusion from gastric vascular ectasias [49]. Also, male sex was predictive of bowel pseudo-obstruction [50] and severe GIT dysfunction [51]. In contrast, other studies found that females more frequently than men had GIT involvement [26], gastroesophageal reflex disease (GERD) [24], and increased risk for GIT progression [52].

In a systematic review and meta-analysis, there was no sex difference for SSc-associated small intestine bacterial overgrowth (SIBO) [53], for lack of esophageal contractility [54], and for bowel scintigraphy findings [55]. These differences may be related to differences in patient recruitment.

### Interstitial Lung Disease

Interstitial lung disease (ILD) is a major cause of morbidity and mortality in SSc patients, since 38% of patients with new diagnosis of SSc-associated ILD (SSc-ILD) develop progressive pulmonary fibrosis (PPF) within 12 months [56].

Most studies have shown an increased frequency of SSc-ILD in men compared to women. Men had ILD more frequently than women in different ethnic SSc populations [26, 28, 30, 57, 58]. In the international EUSTAR database of patients with early SSc, men more frequently than women had ILD and active disease [22]. In two RCTs, men with SSc-ILD had a less favorable course of ILD and worse long-term survival than women [59], and male sex was included in a nomogram to predict PPF, albeit with modest discrimination (C-index 0.72) [60]. Male sex was also associated with faster progression of ILD compared to female sex [61] and was a prognostic factor for increased risk and fast yearly decline in percent-predicted forced vital capacity (FVC) in a large SSc-ILD cohort with long-term observation [62]. In the EUSTAR registry of SSc-ILD patients with multiple FVC measurements over a mean period of 5 years, the strongest predictors for FVC decline were male sex, modified Rodnan skin score (mRSS), and reflex/dysphagia symptoms [63]. However, no sex bias for ILD within 5 years from SSc onset was found in a USA multicenter registry [64].

### Heart Involvement

In SSc, male sex was associated with worse cardiovascular (CV) outcomes, even after adjusting for clinical characteristics [65]. Male patients compared to female patients had more severe heart disease [25], more impaired left ventricular (LV) global longitudinal strain [65], higher frequency of reduced (< 50%) LV ejection fraction (EF), and conduction blocks [27, 58]. However, there was no sex difference for heart failure in a nationwide study in Denmark [66]. Male sex was associated with arrythmias in SSc in a nationwide study in Sweden [67] and was independently associated with major adverse cardiovascular events (MACE) (adjusted HR, 2.0) in the Taiwan Nationwide Health Insurance Research Database [68]. Male sex was also associated with increased incidence of LV diastolic dysfunction in the French National SSc database [69] but not in Chinese patients [70]. It should be noted that the Framingham risk score and the American College of Cardiology/American Heart Association (ACC/AHA) score underestimate CV risk in SSc patients [71].

Most studies found male sex predominance in pulmonary arterial hypertension (PAH). Male sex was associated with a short time from SSc onset to PAH diagnosis [72] and was a predictor for SSc-PAH [73]. In the EUSTAR database with 9182 patients, male sex was independently associated with pulmonary hypertension (PH) (OR 3.0) [20] and was predictive of new-onset PAH (HR 2.66) and heart failure (HR 2.22) [20]. Also, in Thai patients with early SSc, males had a higher frequency of right ventricular dysfunction [29]. However, no sex difference for SSc-PAH was found in a multicenter study from the Mass General Bringham hospitals [26]. In contrast, males were less likely to have PAH in the Canadian SSc research group registry [57].

NFC, as a direct visualization of microvessels, was examined in relation to SSc-PAH. Avascular score in NFC correlated with mean pulmonary arterial pressure in SSc-PAH patients [74] and was independently associated with coronary microvascular dysfunction [75]. Interestingly, male gender in SSc was associated with avascular areas in NFC [76]. However, in a systematic review, sex did not appear to influence the degree of microangiopathy, as detected by NFC in SSc [38].

### Scleroderma Renal Crisis

Scleroderma renal crisis (SRC) occurs in 1–14% of SSc patients with the least frequency in Japan and occurs mostly in early dcSSc. Males had an increased frequency of SRC in the multicenter Italian SSc registry [27] and in Greek patients with early SSc [33]. Also, male sex was a risk factor for SRC in the international EUSTAR cohort with early disease [77]. In SSc-PAH patients, men had an increased frequency of SRC [72]. However, no sex difference in the risk for SRC was found in the German network for SSc [78] and in a multicenter study from the Mass General Bringham hospitals [26].

### Cancer

In a Danish nationwide registry, standardized mortality rate (SMR) for cancer was increased in SSc patients, with men having slightly higher risk than women [79], but in a single-center SSc cohort, cancer was not associated with gender [80]. Other studies found that male sex was associated with lung cancer in patients with SSc [81] and patients with SSc-ILD [82].

### Serology

Male sex was associated with ATA and ARPA, whereas ATAs were the main factor associated with incident DUs in the EUSTAR database of patients with early disease [21]. ATAs were more frequent in men than women in two large SSc cohorts, the Leiden combined care in SSc (CCISS) cohort in Netherlands and the international EUSTAR cohort [22, 83], and also in a large USA single-center SSc cohort [25]. On the other hand, ACAs, which are protective for ILD [84], were more frequent in women [25, 80]. In a systematic review and meta-analysis, anti-TRIM21(Ro52) autoAbs were associated with female sex (HR 1.6) [85]. In patients with early SSc, men had more frequent elevated C-reactive protein (CRP) and active disease than women [22].

### Treatment

There is sparse data on sex differences in response to treatment in SSc. A post hoc analysis of two RCTs for SSc-ILD showed that men had worse course with and without treatment [59]. However, there are other examples of sex bias in response to treatment. Remission in rheumatoid arthritis, defined by strict criteria (no swollen joint, no tender joint, and normal erythrocyte sedimentation rate), was significantly lower in women than men treated with conventional disease-modifying antirheumatic drugs (DMARDs) [86]. Also, male patients responded better to immune checkpoint inhibitors than female patients with cancer [87].

Adverse reactions to medications were reported more frequently in women. In patients with CTD-ILD receiving the antifibrotic nintedanib, liver enzyme elevations and drug interruptions were more frequent in females than males [88]. Pooled data from four RCTs in CTD-ILD treated with nintedanib showed that female patients were more likely to have nausea, vomiting, elevation of aspartate aminotransferase, and, in addition, dose reduction and at least one treatment interruption [88]. However, there was no sex difference in nintedanib discontinuation due to adverse effects [88–90].

A multicenter study of autologous hematopoietic stem cell transplantation (AHSCT) in SSc patients with median follow up of 4.6 years showed that male sex was associated with more events and treatment-related mortality [91]. Also, after lung transplantation, female sex in conjunction with PAH was associated with threefold decreased survival compared to male sex [92].

### Prognosis

The prognosis for SSc remains far from being satisfactory. In a meta-analysis of studies between 1985 and 1996, standardized mortality rate (SMR) for SSc was found to be 1.5–7.2 (95% CI) [93] and was particularly increased in dcSSc [94, 95]. SMR was higher in males compared to females in large SSc cohorts and population-based SSc Registries [94–98]. However, two studies, including a population-based cohort, found that SMR was higher in females than males (4.6 vs 3.2) [99, 100], whereas one study found no significant sex bias for SMR [101].

Male sex was a risk factor for increased mortality in large SSc cohorts from many different countries [94–97, 102–107]. SSc-related mortality [33, 97] and all-cause mortality in SSc were higher in men than women [108]. Smoking was a risk factor for mortality (HR 1.63) in men but not in women with SSc [109]. The increased male mortality in SSc was extended along the course of the disease, i.e., at 1 year [28], 3 years [28], 5 years [20, 25, 28], 8 years [110], and 10 years of follow-up [25]. Male sex was associated with 5-year mortality in early dcSSc (< 2 years from first symptom) [111] and in early disease within 5 years from disease onset/diagnosis (OR:1.93) [112, 113]. However, data from the National Patient Registry in Sweden, which covers hospitalizations and outpatient care, showed no sex bias for the 1-, 5-, and 10-year mortality in SSc patients [114].

Many studies found that male sex was an independent predictor of mortality in SSc. In a prospective 10-year study in the combined care for SSc cohort in the Netherlands, and in the international EUSTAR cohort, male sex was the most important risk factor for all-cause mortality in SSc after adjusting for age, race, and autoantibody status [83]. In a EUSTAR cohort with 9182 patients, after a mean follow up period of 4.9 years, male sex was predictive all-cause mortality (HR 1.48) but not for SSc-related mortality [20]. Also, male sex was an independent risk for mortality in other SSc cohorts including early disease [96, 115–117] and was the main predictor of mortality (HR:2.76) in a multicenter Italian cohort [118] and was included in a prediction rule for 5-year mortality in dcSSc patients [111]. However, male sex was not an independent risk factor for mortality in the Spanish network for SSc registry [119] and was not a predictor factor for mortality in Japanese patients [120].

Internal organ involvement affects mortality differently between sexes. In an early systematic review and meta-analysis of 22 studies with 2244 patients with SSc between 1960 and 2012, male sex was a prognostic factor in SSc-PAH patients [121], and in more recent studies, male sex was a risk factor for mortality in SSc-PAH [122], conferring an HR 2.0–3.9 [123, 124], in SSc-PH [125], in SSc-ILD [124, 126, 127], and in hospitalized patients with connective tissue disease (CTD)-ILD [128]. However, complete right bundle block predicted a higher risk of mortality (HR 5.3) independent of age and sex [129]. A summary of sex differences in clinical aspects of SSc is shown in Table 1.

**Table 1:** Systemic sclerosis: sex bias in clinical manifestations and prognosis

Clinical feature	Main sex differences (Reference)
Age at disease onset	Men are ~ 10 years older than women (15)
Occupational exposure	Male sex is a risk factor (16–19)
dcSSc	Male sex is associated with dcSSc (20–30)
Digital ulcers (DUs)	DUs are more frequently in men (20, 24, 27, 31, 33–36) but no sex difference in the Canadian SSc registry (37)
Musculoskeletal involvement	Arthralgias/arthritis are more frequent in females (24, 39, 41) Myositis is more frequent in males (22, 25, 29, 41) but not in all studies (42, 43)
GIT involvement	Sex bias is controversial, with male predominance (28, 50, 51), female predominance (24, 26, 52), or no difference (53, 54)
Interstitial lung disease (ILD)	Male sex is associated with ILD (22, 26, 28, 30, 57, 58) and progressive lung fibrosis (60–63)
Heart manifestations	Male sex is associated with worse outcome (20, 25, 27, 65), MACE (68), arrhythmias (67), LV diastolic disfunction (70) No sex bias in heart failure (66)
Pulmonary arterial hypertension (PAH)	Male sex is predictor of PAH (20,73) and PH (20) although others found no sex bias (26) or male sex less likely to develop PAH (57)
Scleroderma renal crisis(SRC)	Male sex association (27, 33, 77) OR no sex difference (26, 78)
Serology	ATA associated with males (21,22,25,83) ARPA associated with males (21) ACA associated with females (25,84) Anti-Ro52 antibodies associated with females (85)
Cancer	Male sex was associated with cancer (79–81)
Prognosis	SMR was higher in males (94–98), in females (99,100) or no difference (101) Male sex was a risk factor for mortality (20, 25, 28, 94–97, 102–107, 110,112,113), but not in all studies (114) Some studies found male sex to be predictor of mortality (20, 83, 96, 111, 115–118) but not others (119, 120)
Treatment	Men had worse course of ILD (59) Adverse effects of nintedanib: no sex difference (88, 89) but females had more liver enzyme elevations (88, 90)

*ANA* antinuclear antibodies, *ACA* anticentromere antibodies, *ARPA* anti-RNA polymerase II antibodies, *ATA* anti DNA isomerase I antibodies, *dcSSc* diffuse cutaneous systemic sclerosis, *GIT* gastrointestinal track, *MACE* major adverse cardiovascular event

## Biological Aspects-Sex Differences and Impact on Systemic Sclerosis

Apart for sex chromosomes, females differ from males in reproductive organs and sex hormones. Females have XX chromosomes, and ovaries that produce estrogens, whereas males have XY chromosomes and testes that produce androgens.

### Sex Hormone Differences

Generally, estrogens have immunostimulatory effects whereas androgens have immunosuppressive effects. For instance, estrogens increase immunoglobulin (Ig)-producing plasma cells, stimulate prolactin and interleukin (IL)−6 production, and enhance toll-like receptor (TLR)7-dependent interferon (IFN)α production by plasmacytoid dendritic cells (pDCs) [130–132]. Estrogens also affect immune cells through peroxisome proliferator-activated receptor-γ (PPARγ) which induces profibrotic macrophage 2 (M2) differentiation, inhibits anti-fibrotic Th1 cells, and reduces T follicular helper cells [14]. In contrast, androgens inhibit IL-1β and IL-6 and peripheral blood mononuclear cell activation [133–135]. Sex hormones also greatly affect the expression of autoimmune regulator (*AIRE*) gene, which play a crucial role in immune tolerance [136]. Sex hormones exert differential epigenetic effects, as estrogens promote DNA demethylation and androgens promote DNA methylation. They also have differential effects on microRNAs (miRNAs) and perhaps on gut microbiome [14, 137].

The overall effects of estrogens in SSc appear to be profibrotic, and men with SSc had increased levels of estradiol, whereas women with SSc tended to have lower levels of androgens [138].

### Sex Chromosome Differences

The X chromosome contains around 1100 protein-coding genes, and some of them are associated with immune functions, including *FOXP3* (a master regulator of autoimmunity), *TLR7*, and *TLR8* (involved in innate immunity); cytokine receptor genes (*IL13RA*, *IL9R*, *IL2RG*); *BTK* (encoding Bruton’s tyrosine kinase); *CD40LG* (encoding CD154); *CXCR3*; *IRAK1* (encoding interleukin 1 receptor- associated kinase 1); *TASL* (*CXorf21*, encoding type I IFN response); *KDM6 A* (encoding lysine demethylase 6 A, the epigenetic regulator UTX); and many microRNAs [8].

**Fig. 1:**
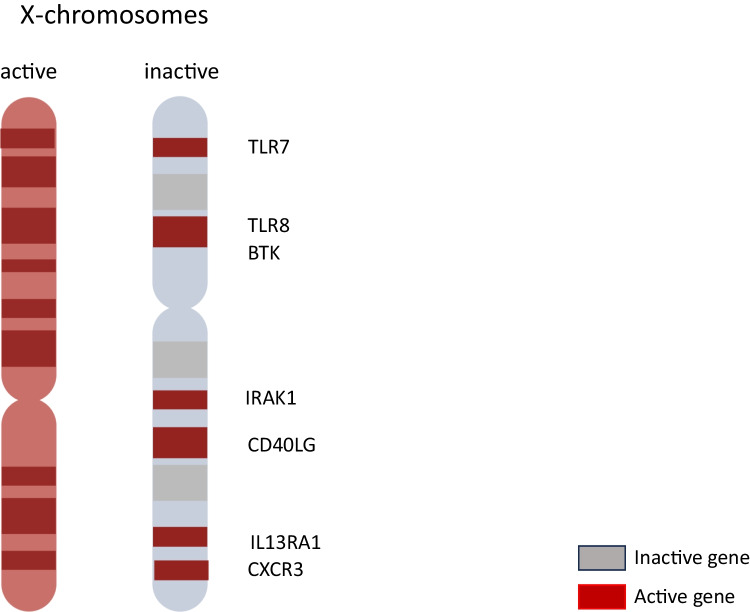
The two X chromosomes in females, active (in red) and inactive (in gray). Genes escaping X-chromosome inactivation in the inactive X chromosome are depicted in red (it has been prepared using BioRender under license specifically for this journal)

The double dose of X-chromosome genes in females is avoided by a random inactivation of one X chromosome (XCI). XCI occurs via (a) upregulation of *Xist* (X-inactive specific transcript), a long non-coding RNA, transcribed from the future inactive X chromosome during embryonic development (present only in females); (b) DNA methylation; and (c) chromatin modification. However, many X-linked genes escape XCI, resulting in biallelic expression of these genes in various cell types, including immune cells [8]. In fact, 15–23% of X-linked genes escape XCI in humans but only 3% in mice [139]. Cytogenetic analysis of cultured lymphocytes from female patients with SSc and female controls revealed that triple X cells were more frequent in female patients [140]. Also, skewed XCI was detected in 44.9% of females with SSc compared to 8% of female healthy donors (HD), and extremely skewed XCI (> 90%) was detected in a third of female patients compared to 2.4% of female HD [141] (Fig. 1).

### Sex Bias in Immune Cells

In the peripheral blood of healthy human beings, CD4 + T cells were higher in young and older women compared to respective men, whereas B cells were higher in older women compared to older men. In addition, with aging, innate cell genes were activated, and T cell genes were inactivated in both men and women, whereas B cell genes were inactivated only in men [142]. Sex bias was also found in human neutrophiles, as RNA sequence and mass spectroscopy of neutrophils from HDs revealed sex differences in mRNA and proteins in neutrophiles [143]. Neutrophil activity is linked to extracellular matrix (ECM) biology, as there is a strong female bias in the neutrophile expression of many extracellular matrix–related genes [144], whereas neutrophils from men compared to those from women exhibit more production of elastase, a serine protease that breaks down elastase and collagen [145]. RNA and assay of transposase accessible chromatin with sequencing (ATAC-seq) profiling of immune cells in female and male mice revealed differentially expressed genes only in macrophages, and these genes were for innate immune pathways [146].

In human T cells, more X-linked chromatin accessible sites were detected in females versus males, as assessed by ATAC-seq [147]. Higher frequency of XCI was found in patients with SSc, and profound XCI skewing was associated with impaired regulatory T cell-suppressive activity [148]. Data from single-cell RNA sequencing and single-nucleotide polymorphisms (SNPs) of B cell subtypes from a female HD revealed that 38 of 53 (72%) X-linked immunity-related genes showed a biallelic expression [149]. In addition, there was a B cell subtype-specific XCI escape with higher levels of biallelic expression in memory B cells and plasmablasts compared to naive B cells and transitional B cells [149].

Single-cell analysis revealed that *TLR7* escapes XCI in B cells, monocytes, and pDCs in women but also in men with Klinefelter syndrome (XXY) [8, 150]. B cells with biallelic *TLR7* expression exhibited increased IgG class switch upon TLR7/T cell stimulation [150]. Variable expression of *TLR7* and *BTK* was found in naive B cells and plasmablasts [149]. BTK plays a key role in B cell antigen receptor (BCR) signaling and is regarded as an important therapeutic target in autoimmune diseases [151]. Of interest, lysosome-associated membrane glycoprotein 2 (LAMP2), a lysosomal protein involved in intracellular antigen presentation, exhibited a biallelic expression in memory B cells and plasmablasts but a monoallelic expression in naive B cells and transitional B cells [149]. Also, IRAK1, mediating IL-1-induced NF-κB upregulation, exhibited a biallelic expression in memory B cells and plasmablasts [149]. IRAK1 mRNA and protein were also increased in cord blood of female neonates compared to that of male neonates [152]. *IRAK1* escapes XCI in SSc patients, as *IRAK1* mRNA levels were higher in female than male patients [153]. It should be noted that an *IRAK1* haplotype that contains a functional variant (rs105972) was associated with dcSSc, ATA, and SSc-ILD [154].

B cells appear to have more influence on innate immunity in women than men, as shown by IRF5, a transcription factor for TLR/My88-mediated response. B cells producing interferon regulator factor (IRF)5 were increased in women than men and produced higher levels of TNFα upon TLR9 stimulation [155]. In a meta-analysis of genome-wide association (GWA) studies IRF5rs2004640, SNP was associated with lung fibrosis in SSc [156], although a later large single-center study did not find IRF5 SNP to be a risk factor for SSc-ILD.

Unstimulating pDCs with a biallelic expression of *TLR7* expressed high TLR7 mRNA and higher IFNα/β mRNA levels [157]. Also, pDCs from females produced more IFNα in response to TLR7 ligands than pDCs from males, resulting in stronger activation of CD8 + T cells [158]. It should be noted that the IRF5, a central mediator of TLR7 signaling [159], was regulated by estrogen receptor 1 gene and was overexpressed in pDCs from females [160]. In addition, its levels positively correlated with pDC IFNα production [160]. Apart from *TLR7*, *BTK* and *IL13RA1* genes variably escape XCI in pDCs, which express higher levels of IFNα [157]. *TLR8* escapes XCI in monocytes and CD4 + T cells [161], and *CD40LG* and *CXCR3* genes were found to escape XCI in T cells [162]. *CD40L* expression was found to be increased in CD4 + T cells from female patients with SSc, and the methylation of DNA regulatory elements in CD4 + T cells was decreased on the inactive X chromosome in female but not in male patients with SSc [163].

The biallelic expression of *TLR7*, and particularly of *TLR8*, appears to have pathogenic consequences in SSc. *TLR7* and *TLR8* expressions were elevated in SSc skin and myoblasts, and levels of TLR8 correlated with skin score [164]. *TLR7* and *as* were found to escape XCI at higher frequency in female SSc patients compared to female HDs [165], and stimulation of TLR7 or TLR7/TLR8 significantly increased type I IFN (IFN-I) expression in pDCs of patients with SSc [166]. It should be noted that IFN-I is important in SSc pathogenesis, detected in SSc before overt fibrosis [167]. Also, stimulation of TLR8 significantly increased tissue inhibitor of metalloptoteinate-1 (TIMP-1) in monocytes of SSc patients and inhibited matrix metalloproteinae-1 activity [168]. In addition, TLR8 promoted inflammation and fibrosis in SSc skin, as overexpression of TLR8 upregulated IL-6, IL-1β, and collagen in skin fibroblasts [164]. Also, TLR8 overexpression increased pDC skin infiltration and fibrosis in mice [169], whereas TLR8 inhibition, but not TLR7 inhibition, prevented fibrosis and alleviated skin fibrosis in established disease in the mouse model of bleomycin (BLM)-induced fibrosis [164, 169]. Peripheral blood pDCs from SSc patients expressed increased levels of TLR8 mRNA and secreted TLR8-dependent CXCL4 (platelet factor 4) [169], although a recent study reported TLR8 production by monocytes, but not by pDCs of patients with SSc [166]. Of note, CXCL4 produced by pDCs forms complexes with DNA/RNA that induce pDC IFNα production, whereas anti-CXCL4 antibodies correlate with type I IFN signature in SSc [170, 171].

A systematic review on single-cell analysis from SSc-ILD found that both macrophages and cytotoxic T cells exhibited increased expression of type I IFN [172]. Interestingly, bronchoalveolar lavage (BAL) from women with SSc-ILD exhibited increased concentration of proinflammatory mediators (G-CSF, IL-12), whereas BAL from men with SSc-ILD exhibited increased concentrations of profibrotic mediators (MMP-13, TIMP 1) [59]. These BAL differences apparently have pathophysiological implications, as men, compared to women, have worse rate of decline of percentage predicted FVC, worse radiographic progression, and increased mortality (HR 2.42) [59].

The XIST ribonucleoprotein (RNP) itself may be a source of autoAbs in SSc, as autoAbs against multiple components of the XIST RNP complex were detected in SSc [173].

### Sex Bias in Autosomal Gene Expression

Apart from sex chromosome genes, there is sex dimorphism in the expression of autosomal genes. Analysis of gene expression in peripheral blood immune cells from HDs revealed that 1553 protein-coding transcripts, of which 93% were autosomal transcripts, were differentially expressed in women compared to men and 72% of these transcripts showed a remarkable sex-bias in only a single immune cell type [174]. Furthermore, female-biased transcripts for each immune cell type were in genes encoding interferons and pattern recognition receptor pathways [174].

Bone marrow neutrophils from mice showed a female bias in the expression of genes throughout the genome [144]. C4 A and C4B encode proteins with distinct affinities for their targets. The copy number of each gene confers differential protection from SSc in men and women. C4 A copy number in men and by C4B copy number in women contributed to stronger protection from SSc [175].

### Genomic Differences

**Fig. 2:**
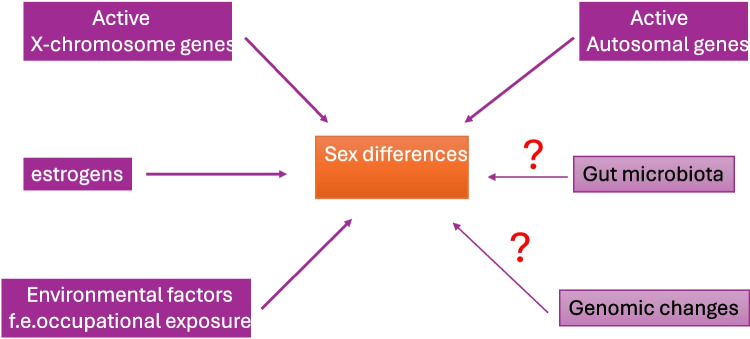
Factors that may influence sex bias in systemic sclerosis encompass a range of elements, including but not limited to activated genes on the X chromosome, active autosomal genes, estrogens, environmental influences such as occupational exposure, and, to some extent, the impact of gut microbiota and other genomic changes

A large genome-wide association (GWA) study in abstract form, examining sex differences, found a locus in the vicinity of neuropilin 1(NRP1) with a suggestive increased risk effect for males and reduced risk for females [176]. Decreased expression of NRP1 was associated with peripheral microvasculopathy, defective angiogenesis, and DUs in SSc patients [177]. In the best-fitting SSc genomic risk score with 33 SNPs in SSc patients of European ancestry, there was no significant contribution of sex, a finding attributed to selection of sex-matched controls [178] (Fig. 2).

## Discussion and Concluding Remarks

It is generally accepted that environmental factors and genes contribute to developing autoimmune rheumatic diseases, including SSc. Although female patients are more susceptible to SSc, male patients have increased morbidity and mortality compared to female patients. This could be explained by the fact that male patients with SSc have increased age at disease onset, more frequently dcSSc, and ILD, risk factors associated with poor outcome.

In mice, sex bias in disease expression was found to be dependent on genetic background but also on strain, as was demonstrated in inflammatory arthritis in different mouse strains [179]. This raises the intriguing possibility for sex bias in disease manifestations in different ethnic groups. Furthermore, the role of sex in gut dysbiosis in SSc remains speculative at present [180]. Sex-linked differences were associated with gut microbiome in C57BL6/J mouse osteoarthritis model, and these differences could be transferred by microbiome transplantation [181]. This might have therapeutic implications for SSc, as fecal microbiota transplantation has already been used in a phase II trial of patients with SSc and bowel symptoms [182]. Longitudinal studies of GIT microbiome in early disease will determine if microbiota alterations will have any impact on disease evolution. A phase I/II RCT of a dual TLR7/TLR8 antagonist in systemic lupus erythematosus suppressed IFN-I signature, showed encouraging efficacy signals, and was well-tolerated [183], and we anticipate that this will be tried in SSc.

It is concluded that that there is sex bias in incidence and severity of SSc, which may be more complex than previously recognized. Therefore, the study of sex bias warrants further investigation in this complex disease. This will facilitate the development of practical algorithms for disease prediction and optimal therapies using artificial intelligence tools.

## Data Availability

No datasets were generated or analysed during the current study.
